# Improve automatic detection of animal call sequences with temporal context

**DOI:** 10.1098/rsif.2021.0297

**Published:** 2021-07-21

**Authors:** Shyam Madhusudhana, Yu Shiu, Holger Klinck, Erica Fleishman, Xiaobai Liu, Eva-Marie Nosal, Tyler Helble, Danielle Cholewiak, Douglas Gillespie, Ana Širović, Marie A. Roch

**Affiliations:** ^1^ K. Lisa Yang Center for Conservation Bioacoustics, Cornell Lab of Ornithology, Cornell University, Ithaca, NY, USA; ^2^ Marine Mammal Institute, Department of Fisheries, Wildlife, and Conservation Sciences, Oregon State University, Corvallis, OR, USA; ^3^ College of Earth, Ocean, and Atmospheric Sciences, Oregon State University, Corvallis, OR, USA; ^4^ Department of Computer Science, San Diego State University, San Diego, CA, USA; ^5^ Department of Ocean and Resources Engineering, University of Hawai'i at Mānoa, Honolulu, HI, USA; ^6^ US Navy, Naval Information Warfare Center Pacific, San Diego, CA, USA; ^7^ Northeast Fisheries Science Center, National Marine Fisheries Service, National Oceanic and Atmospheric Administration, Woods Hole, MA, USA; ^8^ Sea Mammal Research Unit, Scottish Oceans Institute, University of St Andrews, St Andrews, UK; ^9^ Marine Biology Department, Texas A&M University at Galveston, Galveston, TX, USA

**Keywords:** passive acoustic monitoring, bioacoustics, machine learning, temporal context, robust automatic recognition, improved performance

## Abstract

Many animals rely on long-form communication, in the form of songs, for vital functions such as mate attraction and territorial defence. We explored the prospect of improving automatic recognition performance by using the temporal context inherent in song. The ability to accurately detect sequences of calls has implications for conservation and biological studies. We show that the performance of a convolutional neural network (CNN), designed to detect song notes (calls) in short-duration audio segments, can be improved by combining it with a recurrent network designed to process sequences of learned representations from the CNN on a longer time scale. The combined system of independently trained CNN and long short-term memory (LSTM) network models exploits the temporal patterns between song notes. We demonstrate the technique using recordings of fin whale (*Balaenoptera physalus*) songs, which comprise patterned sequences of characteristic notes. We evaluated several variants of the CNN + LSTM network. Relative to the baseline CNN model, the CNN + LSTM models reduced performance variance, offering a 9–17% increase in area under the precision–recall curve and a 9–18% increase in peak F1-scores. These results show that the inclusion of temporal information may offer a valuable pathway for improving the automatic recognition and transcription of wildlife recordings.

## 1. Introduction

Many animals produce sounds for various purposes such as foraging, establishing territories and attracting mates [1]. Passive acoustic monitoring (PAM) methods are used for monitoring and studying a wide variety of soniferous species. The use of automatic recognition techniques has largely underpinned the successes of PAM undertakings by improving the efficiency and repeatability of big data analytics. Most existing automatic recognition techniques, however, only exploit the discriminatory characteristics inherent in the target signals. Consideration of a signal's context as ancillary data in the recognition system offers the potential to improve its recognition performance [2, pp. 90–91]. A signal's context may include a variety of relevant measurements and observations such as meteorological data, time of day, spatial positioning (possibly estimated with directional receivers or receiver arrays) or records of events in the recent past. In recordings obtained with single omni-directional receivers, the temporal context inherent in species' songs (as patterns of temporal separation between note repetitions) may be exploited to more accurately detect individual notes. In this study, we explored the possibility of incorporating such temporal context within the gamut of an artificial neural network-based recognition system.

Improvements in the robustness of automatic recognition system results offer several benefits. From a staffing perspective, automated systems reduce the workload of analysts and facilitate the analysis of large datasets that would not be feasible to annotate manually. Best practices require some level of validation of automatic detections, and improvements in detector reliability would reduce the time required to verify detections (e.g. [3]), leaving more time for addressing scientific questions of interest. Such robust systems can facilitate a suite of ecological studies, such as call- or cue-based density estimation [4], stock identification [5] or cultural transmission of song [6]. Incorporating temporal context could also help separate concurrent singers (the cocktail party problem [7]). Having better measurements of the variabilities in song characteristics (e.g. number of calls, duration, patterns of repetition) could better assist studies focused on understanding the function of songs.

Increased computational power and an abundance of labelled data have substantially bolstered research in machine learning [8] across many fields of research. Artificial neural networks, specifically deep neural networks (DNNs) [9], have become the state of the art across a variety of problem spaces such as computer vision (e.g. object detection, face recognition, scene classification), human speech processing (e.g. speech recognition, text to speech, speaker recognition) and language processing (e.g. translation, auto-correct, auto-suggest). Advances in machine learning research are increasingly being adopted by researchers in the animal bioacoustics community. DNNs have been used in the automatic recognition of vocalizations of birds [10], primates (e.g. [11]), marine mammals (see [12] for a review), marsupials (e.g. [13]), fishes (e.g. [14]) and insects (e.g. [15]). A class of DNNs called convolutional neural networks (CNNs), which leverage two-dimensional information and are used predominantly for processing image data [9], adapt well for the processing of other two-dimensional image-like data, such as spectrograms of audio data. In bioacoustics, CNNs are used to recognize a wide variety of vocalizations (e.g. [3,16–18]). For echolocation clicks, both one-dimensional (e.g. [19]) and two-dimensional (e.g. [20]) CNNs have been used. Recurrent neural networks (RNNs), another class of DNNs, contain internal state (memory) and are capable of handling sequence inputs. Long short-term memory (LSTM) [21] networks and gated recurrent unit (GRU) [22] networks are popular flavours of RNNs. In specific bioacoustic applications, the performance of CNNs and RNNs have been compared. For example, Ibrahim *et al*. [23] compared CNN and LSTM network models for classifying species of groupers (Serranidae) and observed similar performances between the two models. Shiu *et al*. [3] compared a few architectures of CNNs, and a CNN + GRU hybrid model for detecting North Atlantic right whale (*Eubalaena glacialis*) calls and found that one of the CNN models offered the best results.

We propose to combine the ability of CNNs to detect calls in spectrograms of isolated audio segments with the ability of LSTMs to capture temporal patterns from a sequence of such spectrograms. In prior studies, CNNs and RNNs were combined into what was called a convolutional LSTM deep neural network (CLDNN) by Sainath *et al*. [24] and as a convolutional recurrent neural network (CRNN) by Çakır *et al*. [25]. At a conceptual level, a significant difference between our approach and that of CLDNN and CRNN is that their recurrent components operate on a very short-term context (up to a few seconds) whereas the LSTM component in the proposed architecture operates on a relatively longer-term context (approx. 2 min). With CLDNN and CRNN, the temporal context captured in the inputs to the recurrent component is limited to the confines of a single fixed-size time–frequency input (e.g. spectrogram) processed by the preceding CNN layers. In contrast, the recurrent component in our proposed architecture operates on a sequence of scalars and feature embeddings produced by a pre-trained CNN component corresponding to a sequence of successive fixed-size spectrograms. The problem of vanishing gradients [26] that the recurrent networks of CLDNN and CRNN could face in processing long-duration spectrograms is avoided here by having the recurrent network operate on reduced-dimension representations extracted from successive, short-duration spectrograms. At an implementational level, our approach differs from CLDNN and CRNN in that the CNN and LSTM components in our architecture are independently trained. These differences afford us the ability to handle many tens of seconds of audio, thereby capturing sufficient near-term temporal context without encountering computational resource limitations.

We chose fin whale (*Balaenoptera physalus*) vocalizations to evaluate the effectiveness of the proposed approach, given the highly vocal nature of the species and the ready availability of annotated recordings from prior studies. Fin whales occur in most oceans worldwide [27] and have been extensively studied with PAM in the North Pacific [6,28,29], South Pacific [30], Atlantic Ocean [31–33], Indian Ocean [34], Mediterranean Sea [35,36] and Southern Ocean [37,38]. Their vocal repertoire includes many loud, low-frequency calls, of which the stereotyped 20 Hz pulses are the most studied [39,40]. The 20 Hz pulses are short (approx. 1 s), downswept calls with energy centred around 20 Hz. Male fin whales produce songs containing patterned sequences of 20 Hz pulses (henceforth referred to as notes) [41,42]. The central frequency of the notes varies within a song [35,37]. Based on the inter-note intervals (INIs; temporal separation between successive notes), three broad categories of patterned sequences have been identified: singlets (having a single distinct INI), doublets (having two distinct, alternating INIs) and triplets (having two or more distinct INIs, with one of the INIs repeated multiple times) [40,43,44]. Spectrogram cross-correlation [45] based methods are commonly used to detect fin whale notes [30,33,46]. Other approaches include those based on energy and spectral band characteristics [47] and matched filtering [48]. Garcia *et al*. [49] had limited success in recognizing fin whale notes with independent CNN and LSTM network models, and found that the performance of a comparative decision tree classifier was better.

Here, we first trained CNN models to define baseline detection performance. Then, we trained the proposed CNN + LSTM hybrid models to capture the near-term temporal context prevalent as INIs in songs. The reuse of a base CNN model as the ‘CNN’ component in a hybrid model allowed us to better attribute any performance improvements solely to the added temporal context.

## 2. Material and methods

### 2.1. Acoustic data and annotations

**Figure 1.**
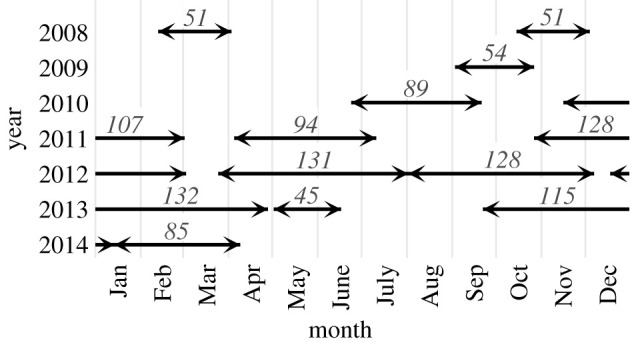
Periods covered by the acoustic data used in the study, with distinct lines (between pairs of diverging arrows) representing different deployments and the numbers over the lines indicating the number of days of data collected during the deployment.

The underwater recordings used in this study were collected as part of prior research (e.g. [28,44]). Data were collected in the Southern California Bight at 34° 19′ N, 120° 48′ W with high-frequency acoustic recording packages (HARPs) [50] over multiple deployments spanning a total of 1210 days (figure 1). The HARPs were configured to record data at a sampling rate of either 200 kHz or 320 kHz. Continuous recordings from the 13 deployments were made available for this study as sets of contiguous audio files. The recording durations covered by each file varied between approximately 10 h and approximately 112 h.

Manual annotation of fin whale notes present in the recordings is detailed in Širović *et al*. [44]. The prior annotation effort focused on labelling only those notes that formed clear song sequences, and any stray notes occurring amidst an otherwise well-structured temporal sequence were not labelled. In an additional round of manual annotations, we labelled these stray notes and added them to the ground truth set. In the third round of manual annotations, we expanded the ground truth set to include representative sections in the recordings where fin whale notes were absent. We reviewed spectrograms of the recordings in Raven Pro 1.6 (https://ravensoundsoftware.com/), and noted the time spans of sections that did not contain any notes. These non-note sections, amounting to an average of 1.43 h per deployment, included periods of silence, shipping noise, other confounding sounds and recording artefacts (equipment noise). These non-note sections constituted the bulk of the negative class samples in the training set.

**Figure 2.**
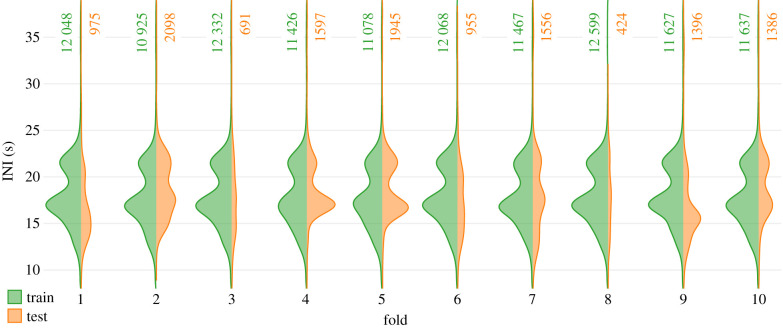
Distribution of inter-note intervals (INIs) in the training and testing splits of each fold. The number of annotated notes available in each split is indicated alongside the respective distributions.

We downsampled all recordings to a fixed sampling rate of 500 Hz to allow consistent processing across different audio files and, in general, to improve workflow efficiency. We randomly partitioned the downsampled audio files from all deployments into 10 disjoint subsets of similar sizes to facilitate the assessment of the training process with a 10-fold leave-one-out cross-validation approach. An arrangement of the 10 subsets into a 9 : 1 grouping constituted a fold, where data from nine subsets formed the training set, and data from the remaining subset were earmarked for testing. The 10 possible ways of constructing a 9 : 1 grouping yielded 10 distinct folds. The randomized partitioning of the full dataset resulted in the assignment of files from a deployment to different subsets, improving the coverage of recording condition variations in the test split of each fold. While it may be possible to lower any risks of producing overly optimistic estimates of absolute model performance by employing other partitioning strategies, the randomized partitioning approach employed here suffices for assessing the relative performance of different models. The distribution of INIs in the training splits were largely bimodal (figure 2), with peaks at approximately 16.9 s and approximately 21.5 s, highlighting the dominance of the short doublet song type in the region [44]. Disparities in the recording durations covered by individual files resulted in test splits of highly variable sizes. Furthermore, the distribution of INIs in a test split did not always resemble that of the respective training split, rendering the test data more challenging.

### 2.2. Input preparation

Recordings in the audio files were split into 4 s segments with an overlap of 3 s (75%) between successive segments. Segment duration and overlap amount were chosen to maximize coverage of notes in the models' inputs such that every note present in a recording was fully contained within at least one segment when processing audio as a continuous stream. Waveforms in the resulting segments were normalized by scaling their amplitudes to the range [−1.0, 1.0], and power spectral density spectrograms (Hann window, 0.8 s frames with 80% overlap; 1.25 Hz frequency resolution) were computed. Spectrogram bandwidth was trimmed to only retain frequencies in the range 10–54 Hz, resulting in two-dimensional surfaces having the shape 36 × 21 (*frequency* × *time*; *height* × *width*). These band-limited spectrograms formed inputs to the CNN model. Spectrograms corresponding to segments that fully contained a ground-truthed note constituted the set of positive class samples. All other spectrograms, including those that contained a note only partially, constituted the negative class sample set.

### 2.3. Neural network architecture

**Figure 3.**
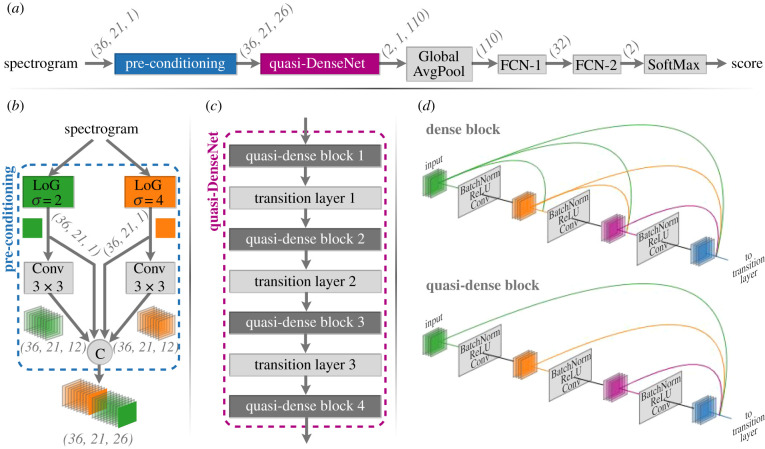
(*a*) Schematic of the base CNN model showing the flow of data along with the shape transformations (in *height* × *width* × *channels* format, where applicable) it undergoes at each layer of the model. (*b*) The spectrogram pre-conditioning layer, comprising one-dimensional Laplacian of Gaussian (LoG) operators applied at two scales, trainable convolution layers (Conv) and a concatenation layer (node C). (*c*) The quasi-DenseNet subnetwork, comprising four quasi-dense blocks. (*d*) Illustration of the difference between a typical dense block (as in DenseNet) and a quasi-dense block. FCN: fully connected network.

**Figure 4.**
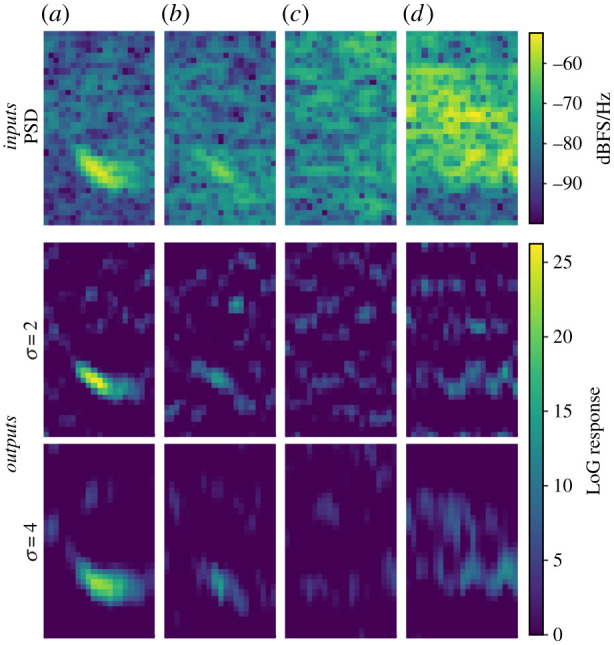
Demonstration of applying Laplacian of Gaussian (LoG) operators. Top row shows power spectral density spectrograms of audio segments containing (*a*) a high-SNR 20 Hz note, (*b*) a low-SNR 20 Hz note, (*c*) ambient noise and (*d*) ship noise. LoG operator responses, restricted to positive values, are shown in subsequent rows.

The architecture of the base CNN model (figure 3) is based on DenseNet [51]. Given that the detection target is a relatively simple and stereotyped signal, we propose a few deviations to the architecture to improve computational efficiency by reducing model size and complexity. Two notable deviations from the classical DenseNet architecture included (i) the use of a custom pre-conditioning layer in place of the first convolution layer and (ii) a reduction in the number of feed-forward connections within a dense block (producing what we reference as a quasi-dense block). The proposed pre-conditioning layer applies one-dimensional Laplacian of Gaussian (LoG) operators at two scales (*σ* = 2, 4) along the frequency axis of the input spectrogram and is followed by the application of twelve 3 × 3 convolutions to the responses at each scale. LoG operators enhance the signal-to-noise ratio (SNR) of features in spectrograms, and the application of multi-scale LoG operators allows spectrographic features of different sizes to be captured in the responses at comparable scales [52] (figure 4). The pre-conditioning layer's outputs, formed from the concatenation of the LoG operators’ outputs and convolution outputs, comprised 26 *channels* (commonly also referred to as *feature maps*). The quasi-DenseNet subnetwork included four quasi-dense blocks with block sizes of 2, 2, 2 and 1 (in that order) and a fixed growth rate of 12. The Global AvgPool layer [53] reduced spatial dimensions (*height* × *width*) by averaging the values in each *channel*. Two fully connected network (FCN) layers were used before the final classification layer.

**Figure 5.**
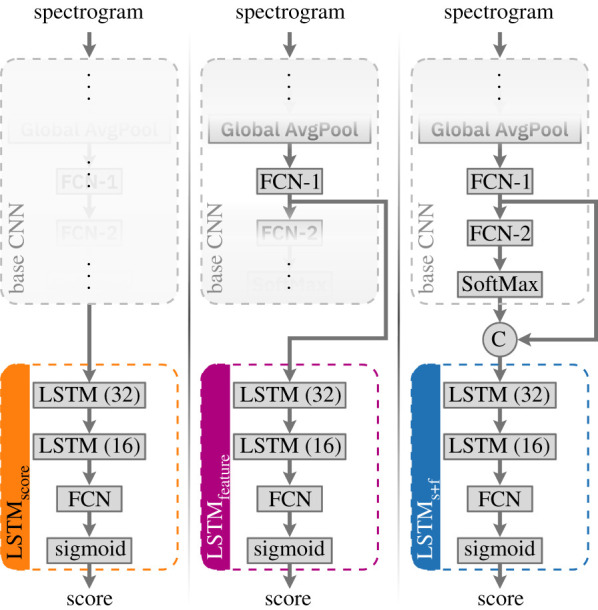
Architectures of the three LSTM variants considered, shown along with the kind of input they operate on. The base CNN model remained the same in all cases; parts thereof relevant for each LSTM variant are highlighted by masking out the other parts.

We considered three variants of LSTM networks for processing temporal context (figure 5). Each variant consisted of a sequence of two LSTM layers with 32 and 16 units (in that order) followed by an FCN layer and sigmoid activation. The first variant, LSTM_score_, operated on the final output (positive class score) of the base CNN model. The second variant, LSTM_feature_, made predictions based on the 32-dimensional feature embedding resulting from the first fully connected network (FCN-1) layer. The final network, LSTM_s+f_ (s + f read as ‘score + feature’), concatenated the score and feature embedding to form a 33-dimensional input vector.

### 2.4. Neural network training

**Figure 6.**
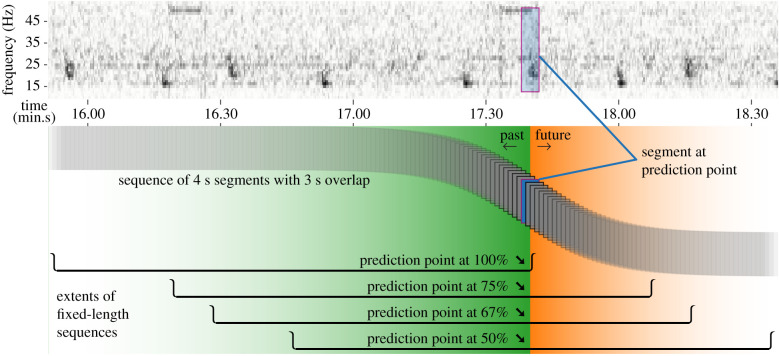
Illustration (bottom row) of how temporal context is encapsulated in the inputs to a hybrid model; shown for an audio clip containing a section of a fin whale song (top row). Each of the stacked rectangles represents a 4 s segment of the spectrogram that gets processed by the base model. Fixed-length sequences of 108 overlapping segments form inputs to the hybrid model. The extents of these sequences, indicated by two-dimensional troughs, show the context available for detecting the occurrence of a note in the highlighted segment. Regions shaded green and orange indicate the past and future contexts, respectively, relative to the highlighted prediction point.

For the base CNN model, the supervised-learning task involved optimizing model parameters to maximize the respective per-class scores (in the range 0–1) produced for input spectrograms from the corresponding classes. Negative class samples that partially contained a ground-truthed note were excluded from the training set while training the CNN model. The base CNN model was trained first, and the same trained model formed part of all the three hybrid variants. Fixed-length sequences of contiguous, overlapping (3 s overlap) spectrograms constituted training inputs for the hybrid CNN + LSTM models. The sequences did not exclude spectrograms with partially contained notes. The length of the input sequence (number of time-steps) quantified the amount of temporal context available to the hybrid models (figure 6). Our choice of 108 time-steps (corresponding to 111 s of audio) ensured coverage of at least three notes within the confines of a song, even for songs having the largest INIs. The learning task for the hybrid models involved optimizing parameters to produce scores close to the binary value (0 for negative class, 1 for positive class) associated with a specific spectrogram in the input sequence of spectrograms. We refer to the spectrogram of interest as the prediction point (PP). In typical detection or classification applications, the PP usually occurs at the end of the input sequence (i.e. at 100% of the considered context) and only captures past events. Shifting of PP to an earlier position in the input sequence allows for incorporating some future context and the position of the PP within the input sequence defines the ratio of past-to-future contexts captured by the input sequence. Our experiments included training hybrid models with the PP set at 100%, 75%, 67% and 50% of the length of the input sequence. The 10-fold cross-validation approach employed resulted in a total of 10 base CNN models and 120 (3 variants × 4 PP settings × 10 folds) corresponding hybrid models.

Training of a hybrid model involved an optimization of just its LSTM subnetwork parameters. This approach is, in essence, a form of transfer learning [54] where the weights of the already-trained base CNN model were frozen (no longer trainable), and the weights of the connected LSTM variant were subsequently initialized and trained independently. As such, training a hybrid model with sequences of spectrograms is the same as training its LSTM subnetwork with equivalent sequences of outputs (scores, feature embeddings or both) from a base CNN model. Furthermore, given that a single base CNN model forms part of 12 corresponding hybrid models, the overall training workflow can be sped up considerably by avoiding repeated execution of the base CNN models. To this end, we applied the trained base CNN model corresponding to each fold to the respective training split and recorded the outputs (scores and feature embeddings). These stored pre-computed outputs were subsequently used to form equivalent input sequences for training the LSTM subnetworks of the hybrid models.

In additional experiments, we assessed the influence of the granularity of time-steps on classification. We reduced the segment overlap amount (see §2.2) from 3 s to 2.75 s. To retain a similar quantity of temporal context, we reduced the number of time-steps from 108 to 88 (which corresponds to 112.75 s of audio). Reducing the number of time-steps not only reduced the number of training samples, but also altered the class prior (the ratio of the number of positive class samples to the number of negative class samples). In addressing this change, we repeated the prior experiments in their entirety, producing another set of 10 base CNN models and 120 corresponding hybrid models.

The training process minimized categorical cross-entropy loss for the base CNN models and binary cross-entropy loss for the LSTM variants using the Adam optimizer [55]. For each fold and model type, the training data were restricted to a maximum of 20 000 samples per class. In all folds, the number of positive class training samples was below this limit (averaging 14 411 for 108 time-steps and 11 412 for 88 time-steps), and the class imbalance was addressed by appropriately weighting the losses during training. Models were trained for 60 epochs (iterations over the full training set), with a randomly chosen 15% of the training spectrograms (spectrogram sequences for LSTM variants) set aside for quick evaluation during the training process. The process of weight initialization and training of models was repeated independently for each of the 260 models.

Models were implemented using TensorFlow 2.0 framework (https://www.tensorflow.org/). They were trained on a Dell Precision 7520 laptop with an Intel Core i7 processor, 32 GB of random-access memory (RAM) and an NVIDIA Quadro M2200 graphics processing unit with 4 GB video RAM. The average per-fold model training durations are presented in table 1.

**Table 1.** Average time (min.s) to train the considered models.

time-steps	CNN	LSTM_score_	LSTM_feature_	LSTM_s+f_
108	25.37	3.22	3.50	3.51
88	23.26	2.39	2.58	3.04

### 2.5. Performance assessment

Recognition performance was quantified with the metrics precision, recall and derived composites, namely precision–recall curve, F1-score and area under the precision–recall curve (AUC-PR). To simplify comparisons, the range of classification scores produced by each model was scaled to the range 0–1 before computing the metrics. The class priors varied among the folds' test splits and differed from those of the full dataset (all 10 subsets considered together). The effects of these variations on perceived performance were suppressed by calibrating [56] the assessments to correspond to the class prior of the full dataset. Calibrated results corresponding to the 10 folds for each model type were aggregated to facilitate making meaningful comparisons between the performance assessments of the different types of models.

## 3. Results

**Figure 7.**
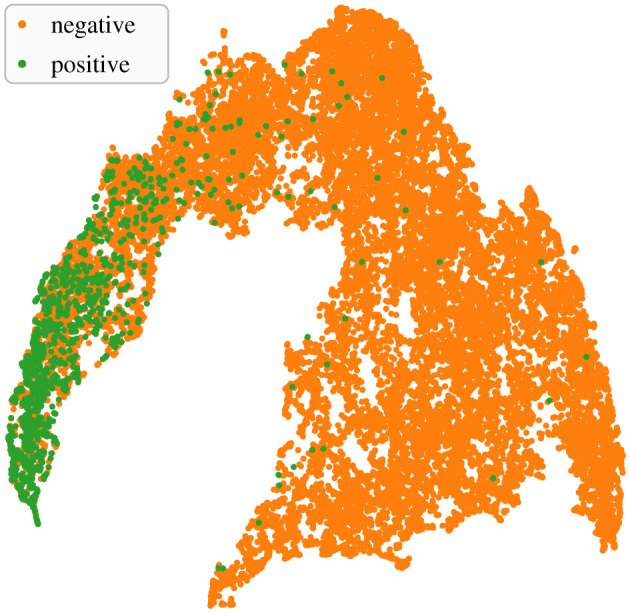
Uniform manifold approximation and projection (UMAP) [57] of feature embeddings in two-dimensional space. The base CNN model considered corresponded to a fold from the experiment with 3 s segment overlap, and the feature embeddings were produced by applying the model to the fold's test split.

**Figure 8.**
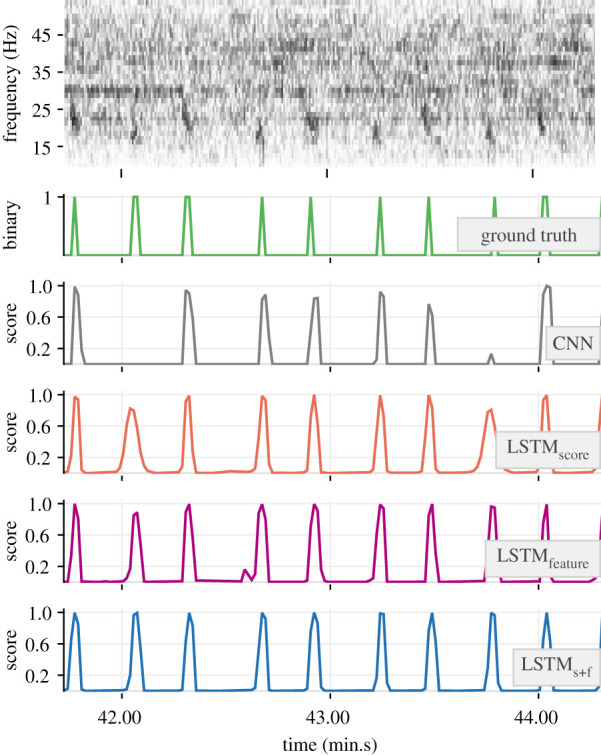
Demonstration of the trained detectors (from the experiment with 3 s segment overlap and PP = 50%) applied to a selected section of a recording. The top row shows the unsegmented spectrogram of the recording. Ground truth values associated with successive segments and the corresponding outputs from each model are shown as connected lines in subsequent rows.

The 32-dimensional feature embeddings output by the base CNN models’ FCN-1 layers encapsulated necessary discriminatory traits of positive and negative class inputs. This was evident in their ability to form *ad rem* clusters in a projected two-dimensional feature space (e.g. figure 7). The outcome of applying the trained detectors on soundscape recordings is demonstrated with a sample section of test data in figure 8. As can be noticed, the base CNN model produced very low scores for the notes at approximately 42.05 and approximately 43.45 (min.s), effectively missing them. Higher scores in the outputs of the hybrid models at these times are indicative of the benefits of incorporating temporal context. Among the hybrid models, the relatively higher scores in the outputs of LSTM_feature_ and LSTM_s+f_ are indicative of higher confidence in their ability to detect such low-SNR notes.

**Figure 9.**
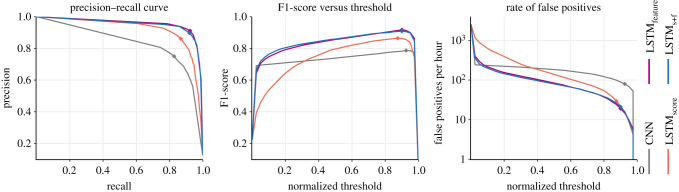
Performance assessment of the base CNN model and the three LSTM variants from the experiment with 3 s segment overlap and PP = 100%. Curves are median values aggregated over 10 folds. Points corresponding to peak F1-scores are indicated with diamond markers.

**Figure 10.**
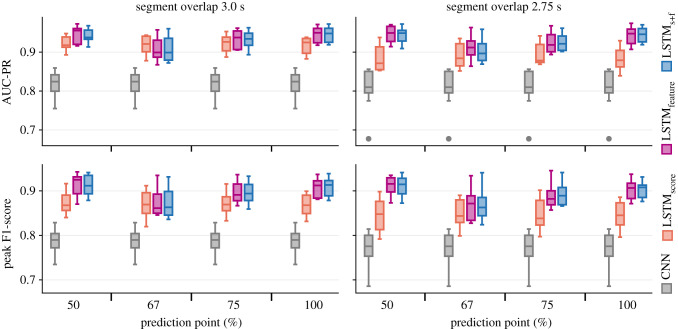
Area under the precision–recall curve (AUC-PR; top row) and peak F1-score (bottom row) as indicators of the performance of the base CNN model and the LSTM variants. The distributions describe the variations in the results across 10 folds for each experiment.

The precision–recall curves indicate that all the LSTM variants offered notable performance improvements over the base CNN model (figure 9; see electronic supplementary material, temporal_context_ESM1.pdf for full results). At moderate-to-high detection thresholds, the hybrid models produced fewer false-positive detections per hour than the base CNN models. Within each experiment, the performance of LSTM_feature_ and LSTM_s+f_ were similar. For easier comparisons, the performance assessments are summarized using peak F1-score and AUC-PR as proxies (figure 10 and table 2). At detection thresholds corresponding to peak F1-scores, LSTM_feature_ and LSTM_s+f_ produced an average of 20.76 false-positive detections per hour of input audio.

**Table 2.** Area under the precision–recall curve (AUC-PR) and peak F1-score as indicators of the performance of the base CNN model and the LSTM variants. Values are means and standard deviations (*µ* ± *σ*) of the respective per-fold quantities from each experiment.

	segment overlap = 3 s				segment overlap = 2.75 s			
	PP = 50%	PP = 67%	PP = 75%	PP = 100%	PP = 50%	PP = 67%	PP = 75%	PP = 100%
AUC-PR								
CNN	0.82 ± 0.03	0.82 ± 0.03	0.82 ± 0.03	0.82 ± 0.03	0.81 ± 0.05	0.81 ± 0.05	0.81 ± 0.05	0.81 ± 0.05
LSTM_score_	0.92 ± 0.02	0.92 ± 0.02	0.92 ± 0.02	0.92 ± 0.02	0.88 ± 0.03	0.89 ± 0.03	0.89 ± 0.03	0.88 ± 0.03
LSTM_feature_	0.95 ± 0.02	0.91 ± 0.03	0.93 ± 0.02	0.95 ± 0.02	0.95 ± 0.02	0.91 ± 0.03	0.92 ± 0.02	0.94 ± 0.02
LSTM_s+f_	0.94 ± 0.02	0.91 ± 0.03	0.93 ± 0.02	0.95 ± 0.02	0.94 ± 0.02	0.90 ± 0.03	0.92 ± 0.02	0.94 ± 0.02
peak F1-score								
CNN	0.79 ± 0.03	0.79 ± 0.03	0.79 ± 0.03	0.79 ± 0.03	0.77 ± 0.04	0.77 ± 0.04	0.77 ± 0.04	0.77 ± 0.04
LSTM_score_	0.87 ± 0.02	0.87 ± 0.03	0.87 ± 0.02	0.87 ± 0.02	0.84 ± 0.03	0.85 ± 0.03	0.85 ± 0.03	0.84 ± 0.03
LSTM_feature_	0.91 ± 0.02	0.87 ± 0.03	0.90 ± 0.02	0.91 ± 0.02	0.91 ± 0.02	0.87 ± 0.03	0.89 ± 0.02	0.90 ± 0.02
LSTM_s+f_	0.91 ± 0.02	0.87 ± 0.03	0.90 ± 0.02	0.91 ± 0.02	0.91 ± 0.02	0.87 ± 0.03	0.89 ± 0.02	0.90 ± 0.02

**Figure 11.**
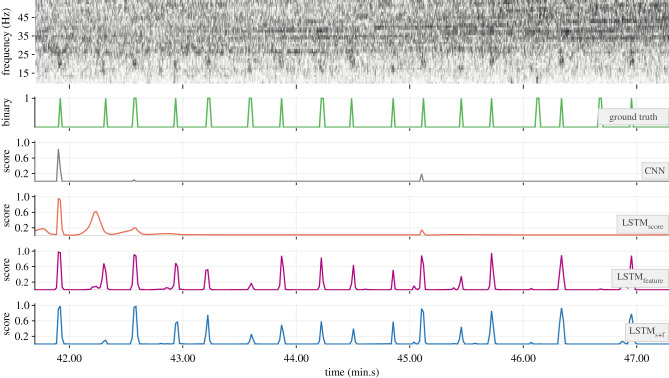
Demonstration of contrasting detection capabilities of the trained models (from the experiment with 3 s segment overlap and PP = 100%) under extreme shipping noise. The top row shows the spectrogram of the recording. Associated ground truth values and the corresponding outputs of each model are shown in subsequent rows.

Much of the observable performance differences (between the base model and the corresponding hybrid models; between LSTM_score_ and the other two hybrid variants) manifested under conditions of low and variable SNR (see electronic supplementary material, temporal_context_ESM2.pdf for a systematic assessment). A representative example (figure 11) shows the base CNN model failing to produce high scores for a bout of low-SNR notes following the first note. Consequently, LSTM_score_ failed to produce high scores beyond the second note, as the causal benefits of learned context continued to fade. Since the intermediate feature embeddings (outputs of the FCN-1 layer) encapsulate more information than the scalar scores output by the CNN model, the cascaded attenuation of the benefits was markedly lower in the case of LSTM_feature_ and LSTM_s+f_.

**Figure 12.**
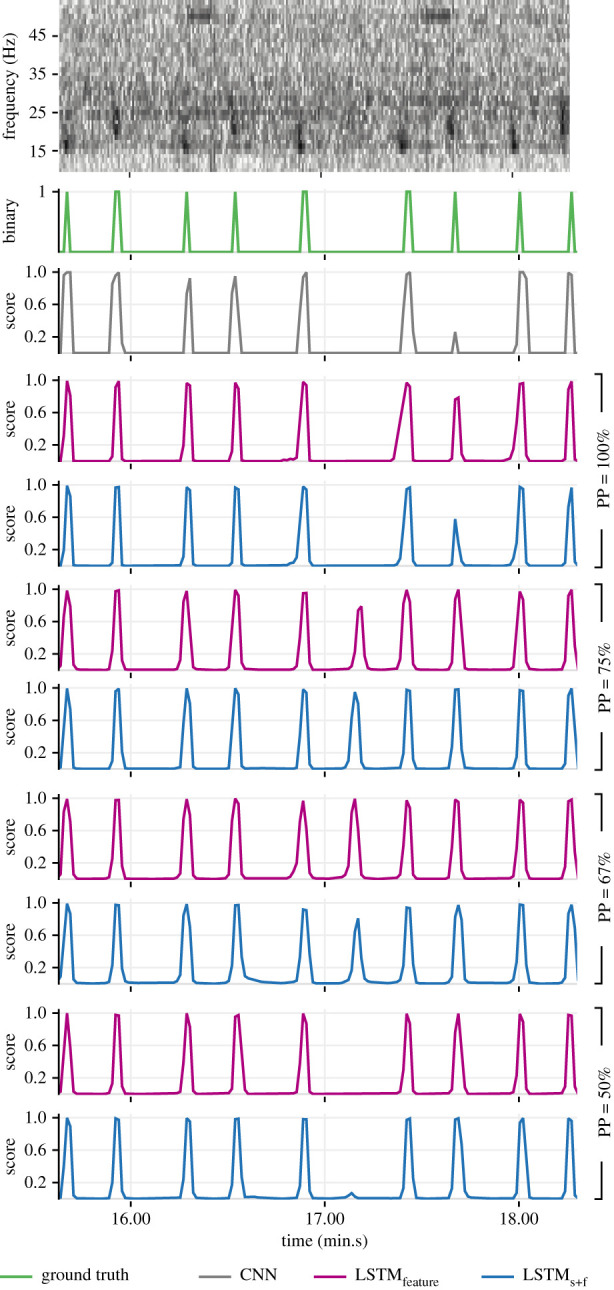
Demonstration of contrasting detection capabilities of LSTM_feature_ and LSTM_s+f_ models (from the experiments with 3 s segment overlap) at different settings of prediction point (PP). The top row shows the spectrogram of a recording with a missing note within a song. Associated ground truth values and the corresponding outputs of each model are shown in subsequent rows.

The performance at PP = 67% and PP = 75% was relatively lower than that at other PPs. This was driven predominantly by a reduced precision in the models' detections. A representative example demonstrates the contrasting abilities of LSTM_feature_ and LSTM_s+f_ at different PPs to avoid producing false positives for missing notes within a song (at approx. 17.14 in figure 12). Such missing notes could be either real (behaviour of the singing whale) or apparent (caused by variable SNR).

**Figure 13.**
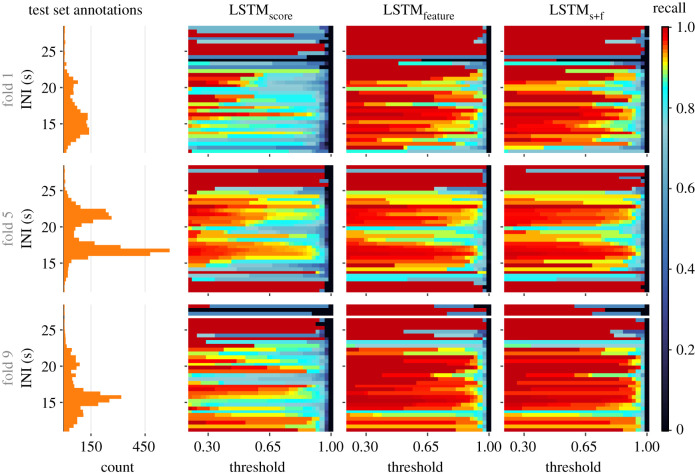
Recall as a function of ground truth notes' inter-note intervals (INIs), for trained hybrid models from the experiment with 2.75 s segment overlap and PP = 100%. Results for the selected three folds represent conditions where similarities in INI distributions among pairs of train–test splits ranged from identical (fold 5) to highly dissimilar (fold 1).

Performances for folds with atypical distribution of INIs in the test splits (e.g. folds 1, 3 and 6; figure 2) were comparable to the general overall performance. Furthermore, we assessed the hybrid models’ recall as a function of the ground-truthed notes' INIs (figure 13; electronic supplementary material, temporal_context_ESM3.pdf). For notes with INIs close to the dominant INIs at approximately 16.9 s or approximately 21.5 s (figure 2), recall remained high at moderate-to-high thresholds across all experiments. This is indicative of the influence of dominant INIs (in the training data) on learning. Recalls at non-dominant INIs were markedly lower for LSTM_score_. By contrast, LSTM_feature_ and LSTM_s+f_ demonstrated a better ability to detect notes at non-dominant INIs, offering high recall even at high thresholds. This difference can also be attributed to the higher degree of information encapsulation in feature embeddings.

Overall, average improvements in AUC-PR, relative to the base CNN model, varied in the range 9–13% for LSTM_score_ and 11–17% for the other two hybrid variants. Peak F1-score improvements averaged 9–11% for LSTM_score_ and 11–18% for the other two hybrid variants. At the respective peak F1-scores, the average number of false-positive detections per hour were (relative to the base CNN model) 56–76% lower for LSTM_score_ and 59–85% lower for the other two hybrid variants. Inter-fold variance in the performance of LSTM_feature_ and LSTM_s+f_ decreased notably when PP was either 100% or 50%, and these models produced the best overall results with average peak F1-scores of 0.91 and average AUC-PRs of 0.95.

## 4. Discussion

We considered the role that temporal context could play in increasing the reliability of annotating bioacoustic signals in soundscape recordings. We extended the ability of a convolutional model to perform classification on isolated audio segments by feeding its outputs into recurrent networks that exploited temporal patterns. The proposed hybrid framework, composed of independently trained CNN and LSTM subnetworks, offered a flexible workflow and processing efficiency. The performance assessments showed that call recognition in isolated audio segments (by a CNN model) improved with the addition of temporal context (by way of attaching a recurrent network to the CNN). Among the hybrid models, those operating on feature embeddings offered better performance than the model that considered only baseline scores. The precision–recall curves of all the hybrid variants dominated that of the base model (figure 9). The hybrid models offered F1-scores higher than the peak F1-score of the base CNN model across a considerable range of detection thresholds. The nature of the observable improvements implied that incorporating temporal context improved the ability both to successfully reject audio segments without a note and to detect notes that a standalone CNN model could miss. The ensuing higher precision and a lower rate of false positives help reduce the burden of downstream validation efforts (in applications such as presence/absence monitoring, density estimation, etc.) [3]. Performance improvements remained consistent across experiments involving different ways of altering models’ inputs, demonstrating the effectiveness of the proposed methodology and framework, and validating our hypothesis that the use of temporal context improves detection of call sequences.

Among the proposed deviations to the DenseNet architecture, the substitution of the initial conventional convolutional layer with the custom pre-conditioning unit was aimed at improving the discriminatory characteristics in the early-to-intermediate features generated within the network. High accuracies attained during training (and validation) may, in part, be attributed to the pre-conditioning unit. The base CNN model and the hybrid models were all subject to similar training schemes (optimizer type, number of epochs, etc.), and no explicit data augmentation techniques were considered. While it may be possible to achieve further performance improvements with architecture-specific tuning of training parameters, the restrictions we considered ensured that the hybrid models' performance gains over the base CNN model could be firmly attributed to the added temporal context. The multi-fold approach allowed us to assess the effects of different initializations of the models’ weights on recognition performance. Consistency in performance across different folds suggested that overfitting and biases from weight initialization were unlikely.

**Figure 14.**
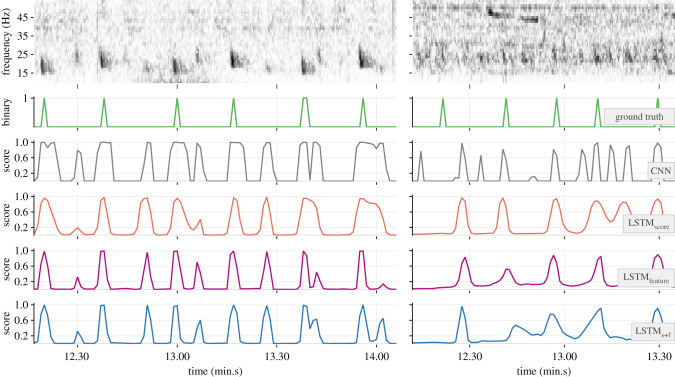
Demonstration of detection capabilities of the trained models in the presence of two concurrent songs (left column; recording from 2013) and many concurrent songs (right panel; recording from 2010). The top row shows the spectrogram of the recording. Subsequent rows show the associated ground truth values and the corresponding outputs of each model.

The multi-year acoustic data considered in the study are expected to have captured any seasonal variations in fin whale singing behaviour. Randomized partitioning of the recordings across folds ensured representation of the variations in the training and test splits of each fold. The annotated dataset contained several instances of fin whale songs where the patterned INIs were readily discernible. The additional round of manual labelling to annotate stray notes ensured that detections corresponding to such notes were not considered false positives and that the reported precision values more closely reflected the true performance of the models. The dataset also contained instances of concurrent songs, ranging from cases where individual notes and INIs were distinguishable to cases where songs from many distant individuals made discerning individual notes (and, hence, the INIs) difficult. The latter case is common during winter breeding seasons [42,58]. Where concurrent songs were present, the annotations only had notes from the dominant foreground song labelled. As such, detector precision appeared to suffer under these conditions (e.g. figure 14). Inclusion of annotations for concurrent songs' notes in the training data could offer further performance improvements. Automated separation of concurrent song tracks may warrant additional elements in the model architecture.

The experiments with different segment overlap amounts were aimed at assessing the impacts of input granularity. Lowering of the segment overlap amount from 3 s to 2.75 s reflected in a marginal reduction in the performance of the base CNN models. Consequently, there were notable reductions in the recognition performance of LSTM_score_. By contrast, the recognition performance of LSTM_feature_ and LSTM_s+f_ remained largely unchanged, hinting at the prospect of improving computational efficiency by increasing input granularity.

The experiments with a shifting of PP were aimed at assessing the impacts of altering the ratio of the quantity of past-to-future contexts. It could be argued that, at PP = 100%, the leading (most recent) features in an input sequence exert greater influence on the models’ outputs. While shifting PP backwards would lower this influence, we, however, anticipated that the availability of both past and future contexts would further improve a model's ability to make accurate detections. However, given that the dataset was not perfect (for example, see the ground truth at approx. 46.40 in figure 11) and that, at PP = 100%, the performance was already quite high (in figure 9, precision–recall curves for LSTM_feature_ and LSTM_s+f_ lie very close to the desired ideal at the top-right corner), there was little scope for further performance improvements. As such, the performance at PP = 50% was only marginally better than that at PP = 100%. On the other hand, performance at PP = 75% and PP = 67% deviated significantly from the trajectory between the performance at PP = 100% and PP = 50%. The durations of future contexts at PP = 100% and PP = 50% (0 s and approx. 55 s, respectively) were well beyond the range of known INIs possible in fin whale songs whereas the durations of future contexts available at PP = 75% (approx. 37 s) and at PP = 67% (approx. 28 s) were within the range of maximum possible INIs in the dataset (figure 2). Skipped notes (approx. 17.14; figure 12) and missed annotations (approx. 46.40; figure 11) occurring around these INIs not only affected performance assessments directly but may have also impacted learning during training, thereby producing the aforementioned performance trajectory deviation.

The high computational complexity involved in training (and using) models that operate on long-duration audio segments (capturing temporal context) was successfully tackled here with a divide-and-conquer approach that combined the abilities of independently trained CNNs and LSTM networks. Handling inputs of approximately 111 s (36 × approx. 690) would be problematic with other composite architectures such as CLDNN and CRNN. Among the proposed deviations to the DenseNet architecture, replacing dense blocks with quasi-dense blocks resulted in a 27% reduction in overall model size (approx. 54 000 parameters total). Given their inherent ability to process audio in streaming mode, these lightweight models are conducive for application in real-time monitoring on resource-constrained PAM equipment.

The parameter settings for input preparation (such as segment duration and bandwidth) and the model architectures (such as depth and width, and PP) were chosen to fit the considered problem domain, i.e. detection of notes in fin whale songs. However, the proposed hybrid architecture and the associated workflow are quite generic. On one hand, the modular characteristic allows one to replace our custom DenseNet with a different type of pre-trained CNN model or to replace the LSTM network with a GRU network. On the other hand, the proposed hybrid scheme and the associated training workflow may be adapted for detecting notes of other singing animals, such as birds, with apposite modifications to audio pre-processing and model construction. One must, however, ensure that the base CNN model is trained well before going on to train a hybrid model. The assessment of the base CNN model may employ an empirical approach (figure 7), a systematic approach or both (as performed in this study). While the problem domain considered in this study was limited to songs containing a single type of note, the note itself exhibited notable spectro-temporal variations. We expect the proposed scheme to offer similar benefits in problem domains that have small to medium-sized note repertoires as well.

## Data accessibility

Implementation of the proposed model architectures, the associated training workflow and evaluation mechanism are available at https://doi.org/10.5281/zenodo.4661565 [59] (https://github.com/shyamblast/TemporalContext-2021). A subset of the downsampled audio files used in this study, along with the corresponding annotations (both primary and secondary), is available at https://doi.org/10.5281/zenodo.4661494 [60].
